# Myocardial strains from 3D displacement encoded magnetic resonance imaging

**DOI:** 10.1186/1471-2342-12-9

**Published:** 2012-04-25

**Authors:** Katarina Kindberg, Henrik Haraldsson, Andreas Sigfridsson, Jan Engvall, Neil B Ingels, Tino Ebbers, Matts Karlsson

**Affiliations:** 1 Department of Management and Engineering, Linköping University, SE-581 83 Linköping, Sweden; 2 Center for Medical Image Science and Visualization (CMIV), Linköping University, SE-581 85 Linköping, Sweden; 3 Department of Medical and Health Sciences, Linköping University, SE-581 85 Linköping, Sweden; 4 Department of Cardiothoracic Surgery, School of Medicine, Stanford University, Stanford, CA 94305, USA; 5 Research Institute of the Palo Alto Medical Foundation, Palo Alto, CA 94305, USA; 6 Department of Science and Technology, Linköping University, SE-581 83 Linköping, Sweden

## Abstract

**Background:**

The ability to measure and quantify myocardial motion and deformation provides a useful tool to assist in the diagnosis, prognosis and management of heart disease. The recent development of magnetic resonance imaging methods, such as harmonic phase analysis of tagging and displacement encoding with stimulated echoes (DENSE), make detailed non-invasive 3D kinematic analyses of human myocardium possible in the clinic and for research purposes. A robust analysis method is required, however.

**Methods:**

We propose to estimate strain using a polynomial function which produces local models of the displacement field obtained with DENSE. Given a specific polynomial order, the model is obtained as the least squares fit of the acquired displacement field. These local models are subsequently used to produce estimates of the full strain tensor.

**Results:**

The proposed method is evaluated on a numerical phantom as well as *in vivo *on a healthy human heart. The evaluation showed that the proposed method produced accurate results and showed low sensitivity to noise in the numerical phantom. The method was also demonstrated *in vivo *by assessment of the full strain tensor and to resolve transmural strain variations.

**Conclusions:**

Strain estimation within a 3D myocardial volume based on polynomial functions yields accurate and robust results when validated on an analytical model. The polynomial field is capable of resolving the measured material positions from the *in vivo *data, and the obtained *in vivo *strains values agree with previously reported myocardial strains in normal human hearts.

## Background

The pumping behavior of the heart consists of complex sequences that constitute cardiac contraction and relaxation. The kinematic behavior of the heart has been analyzed extensively in order to understand the mechanisms that impair the contractile function of the heart during disease. Until recently, the only method with high enough spatial resolution of three-dimensional (3D) myocardial displacements to resolve transmural behaviors was invasive marker technology [1,2]. However, the recent development of magnetic resonance imaging (MRI) methods such as harmonic phase (HARP) analysis of tagging [3] and displacement encoding with stimulated echoes (DENSE) [4], make detailed non-invasive 3D transmural kinematic analyses of human myocardium possible for clinical and research purposes [5].

A previously presented polynomial method for cardiac strain quantification from surgically implanted markers and beads enables straightforward 3D strain computation within the myocardium [6]. For the marker and bead data, this method was shown to yield accurate and robust results, with errors smaller or comparable to a previously presented finite element method tailored for the same type of data, and has been applied to bead displacements for analyses of systolic and diastolic myocardial strains [7-9]. The method is simple in nature which aids to bridge a possible gap in understanding between different disciplines and has specifically been shown to be well suited for sparse arrays of displacement data [6]. The present work applies this polynomial method to 3D MRI displacement data as it: 1) allows for quantification of the full 3D strain tensor; 2) resolves transmural strain variations within the myocardium, and 3) provide robustness to noise. Displacement measurements were acquired within a 3D myocardial slab at the left ventricular (LV) equator. The 3D volume was divided into segments where the polynomial method was applied to quantify the Lagrangian strain tensor. The method was evaluated on a numerical phantom as well as *in vivo *on a healthy human heart.

## Methods

### Strain analysis

Displacement-encoded MRI acquire displacements **u **at time t_n _of timeframe n relative to a reference configuration of the myocardium, at the spatial coordinates **s **= (s_x_, s_y_, s_z_). Lower-case letters are used to denote the coordinates of the deformed myocardium. The spatial coordinates **S **= (S_X_, S_Y_, S_Z_) of the myocardium in the reference configuration were derived by subtracting the displacements from the spatial coordinates at the current configuration; **S**(t_1_) = **s **- **u**(**s**, t_1_). The upper-case letters are used to denote the coordinates of the reference configuration.

**X **= **X**(**S**) is the coordinate of a material point, defined as an infinitesimal volume of myocardial tissue, in the reference configuration and **x **= **x**(**s**) is the coordinate after a deformation of the body. The mapping from reference to deformed configuration was modeled by a polynomial function **g**(**X**) of the coordinate of the material point in the reference configuration [6]. This polynomial position field gives an estimate $\hat{x}$ of the measured coordinate **x **and can be of different order in the different reference coordinate directions, radial (X_R_), circumferential (X_C_), and longitudinal (X_L_), depending on the number of material points along each dimension, and can thus be described as

(1) $$\hat{x}=g\left(X\right)=f_{\text{R}}\left({\text{X}}_{\text{R}}\right)f_{\text{C}}\left({\text{X}}_{\text{C}}\right)f_{\text{L}}\left({\text{X}}_{\text{L}}\right),$$

where **f**_R_, **f**_C _and **f**_L _are polynomial functions of X_R_, X_C _and X_L_, respectively. The Lagrangian strain tensor **E **is a measure of deformation and is connected to the deformation gradient tensor **F **via the definition

(2) $$E=0.5\left(F^{\text{T}}F-I\right),$$

where **I **is the identity tensor. The deformation gradient tensor is given by differentiation, with respect to reference position, of the mapping from reference to deformed configuration, **F **= ∂**x**/∂**X**.

The principal strains E_1_, E_2 _and E_3 _are obtained by diagonalization of the strain tensor, and their magnitudes are independent of any reference coordinate system. Principal strain E_1 _represents maximum lengthening and E_3 _represents maximum shortening.

In the subsequent analytical and *in vivo *evaluation, the 3D volumes were divided into 80 overlapping segments covering the circumference. Each of the segments was 7.5 mm thick and π/6 radians wide between the endo- and epicardial border. Within each segment the material points were transformed to local Cartesian cardiac coordinates with X_C _defined as tangential to the surface of the volume and parallel with the short axis planes, X_L _defined as orthogonal to the short axis planes and oriented apex-to-base, and X_R _defined parallel with the short axis planes, orthogonal to X_C _and oriented outwards from the volume.

Four orders of the polynomial function **g**(**X**) were analyzed. 1) A bilinear-quadratic polynomial (BLQ) was bilinear within the X_C_-X_L _plane and quadratic in X_R_, 2) a bilinear-cubic polynomial (BLC) was bilinear within the X_C_-X_L _plane and cubic in X_R_, 3) a linear-quadratic-cubic polynomial (LQC) was cubic in X_R_, quadratic in X_C _and linear in X_L _and 4) a biquadratic-cubic polynomial (BQC) was biquadratic within the X_C_-X_L _plane and cubic in X_R_. For example, for the LQC polynomial field, the estimated coordinates were given by

$${\hat{x}}_{i}=\left(a_{1i}X_{R}^{3}+a_{2i}X_{R}^{2}+a_{3i}X_{R}+a_{4i}\right)\left(a_{5i}X_{C}^{2}+a_{6i}X_{C}+a_{7i}\right)\left(a_{8i}X_{L}+a_{9i}\right)$$

(3) $$=\left[\begin{array}{ccc} X_{R}^{3}X_{C}^{2}X_{L}, & \ldots , & 1 \end{array}\right]\cdot \left[\begin{array}{c} c_{1i}\\ \vdots \\ c_{24i} \end{array}\right]$$

where *a_ki _*and *c_li _*are sets of constants, *i = R, C, L*, and *k *and *l *are serial numbers for the indices. The polynomial field for each segment was determined by minimizing the squared difference between the measured and the estimated coordinates of the material points belonging to the segmentation.

(4) $$\underset{c_{i}}{\min}\overset{m}{\underset{j=1}{\sum }}{\left(x_{ij}-{\hat{x}}_{ij}\left(c_{i}\right)\right)}^{2},$$

where *m *is the total number of points within the segment and ***c****_i _*are all the constants *c_li_*. The polynomial was evaluated and the Lagrangian strain tensor quantified at material positions along the radial centerline within each segment. The accuracy of the polynomial function was evaluated by the residual of the polynomial mapping, determined as the root-mean-squared difference (RMS) between the measured (**x**) and estimated ($\hat{x}$) coordinates within each segment. The strain analysis was performed off line using Matlab 7.6.0 (The MathWorks, Inc.).

### Analytical evaluation

A previously presented analytical model [6,10] was used to evaluate the strain computation. Briefly, the model was a thick-walled cylinder that deforms to resemble the LV during systole. The cylinder was adapted to the systolic *in vivo *data with inner radius of 28.5 mm and outer radius of 42.4 mm at the reference configuration, and inner radius of 23.6 mm at the deformed configuration. Material points were sampled through the cylinder wall within ten short axis planes with 2.5 mm separation between the planes and 2.5 × 2.5 mm separation between sampled points within each plane.

Strains were computed throughout the model using the method described above. For all polynomial orders, the estimated strains were compared with the analytical strains (*Ẽ_IJ _*, where *I,J *= *R,C,L*). To mimic the acquisition of *in vivo *data, noise was added to the analytical model to investigate the noise sensitivity of the analysis method.

Three normalized distributions of noise were analyzed, each with a mean value of zero and with the standard deviations 0.05 mm, 0.10 mm and 0.30 mm, respectively. The noise levels evaluated were chosen to represent practical measurement errors in DENSE displacement measurements corresponding to SNR in the order of 35, 17 and 6.

### *In vivo *evaluation

*In vivo *evaluation was performed by applying the strain quantification method to displacements acquired by DENSE MRI in a normal human heart, and comparing the results with strains obtained for normal human hearts from other studies.

**Figure 1:**
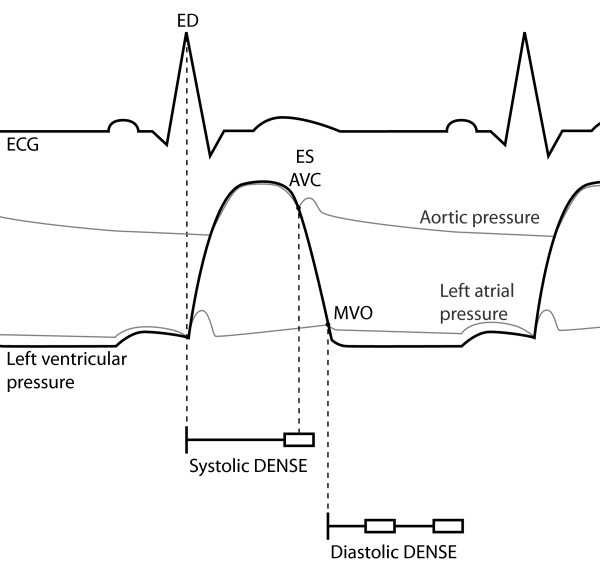
**Timing of acquisition**. Definitions of the time frames of end diastole (ED), end systole (ES), mitral valve opening (MVO) and the two time frames during diastolic filling (75 ms and 213 ms after MVO, respectively). ED is defined at the ECG R-wave peak and ES at aortic valve closure (AVC).

Anatomical images and displacement data were acquired in a 31-year old healthy volunteer on a 1.5 T MR scanner (Philips Achieva, Philips Medical Systems, Best, The Netherlands). Displacement data were acquired, using an in-house DENSE implementation, in three cardiac phases as illustrated in Figure 1; one in systole, and two during diastole related to early and late filling. The cardiac phases were defined from balanced steady state free precession acquisition in a standard 3 chamber view with a temporal resolution of 13 ms. These images were used to define mitral valve opening (MVO) and end systole (ES), defined as aortic valve closure (AVC). These two events were used to time the measurement of displacement in systole and diastole, acquired in 3D short axis slab in two separate 3D DENSE acquisitions. For the systolic measurement, the initial reference position was encoded at end diastole (ED), defined by the ECG R-wave peak, and the displacement was read out at ES, as shown in Figure 1. For the displacement in diastole, the initial reference position was encoded at MVO and read out at both MVO + 75 ms and at MVO + 213 ms, which corresponds to approximately 22% and 62%, respectively, of the LV filling interval. The 3D DENSE acquisitions were performed with an ECG triggered, respiratory navigator gated acquisition with the following parameters: acquisition voxel size 2.5 × 2.5 × 2.5 mm, field of view 350 × 350 × 25 mm, *k*-space segmentation factor 3, EPI factor 7, parallel imaging (SENSE) with reduction factor 2, echo time 5.7 ms, repetition time 12.2 ms, balanced multi-point displacement encoding 0.35 cycles per pixel[11], 3-SPAMM [12] displacement encoding with optimized flip angle [13]. The sequence requires data acquisition of 346 heart beats. Including navigator gating, the scan time is in the order of 10-15 minutes, depending on heart rate and navigator efficiency. For this DENSE sequence, a noise level of about 0.05 mm can be expected for a typical in-vivo measurement.

Segmentation of the myocardium was performed using the freely available software Segment [14]. To avoid that non-myocardial tissue could affect the myocardial strain estimates, the segmentation was conservative, excluding uncertain areas or voxels. The phase of the DENSE MRI is proportional to the myocardial displacement, but can be an arithmetic modulo 2π, which can be represented by wrapping the phase into the interval -π to π. Using the segmentation to outline the myocardium, the MRI phase was unwrapped [15] using a multigrid solver to the Poisson equations [16].

Systolic Lagrangian strains were analyzed at ES, with reference configuration at ED. Diastolic Lagrangian strains were analyzed during LV filling with reference configuration at MVO.

The research has been performed with informed consent, approved by the Regional Ethical Review Board in Linköping and carried out in compliance with the Helsinki Declaration.

## Results

### Analytical evaluation

**Figure 2:**
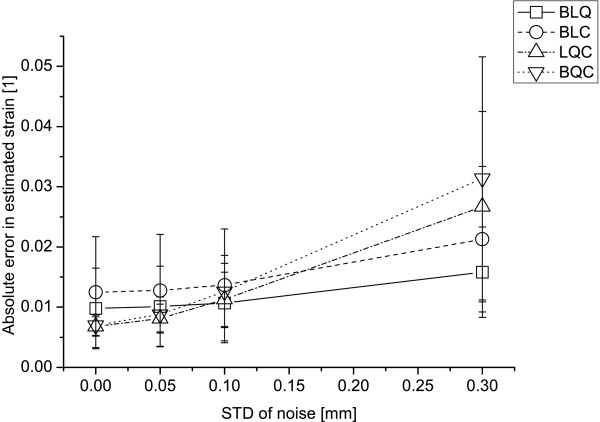
**Sensitivities to noise**. The mean ± SD absolute error in estimated strain of each polynomial for different extents of noise on the analytical model. BLQ: bilinear-quadratic polynomial, BLC: bilinear-cubic polynomial, LQC: linear-quadratic-cubic polynomial, BQC: biquadratic-cubic polynomial.

The size of the errors of estimated strains is dependent on the spatial resolution of the sampled displacements [6]. Given the analytical strain components *E_IJ _*of the model, the absolute errors of the estimated strains in the analytical model, *ε_IJ _*= |*Ẽ_IJ_*-*E_IJ_*|, were computed for each polynomial configuration order, integrated throughout each plane and averaged over the planes. For the noise free case, the mean ± SD error for the strain components of each polynomial order were BLQ: 0.0098 ± 0.0067, BLC: 0.0125 ± 0.0092, LQC: 0.0068 ± 0.0016, BQC: 0.0070 ± 0.0017. The errors for each component, respectively, from the linear-quadratic-cubic polynomial, for which the smallest mean errors were obtained, were ε_RR _= 0.0081 for the radial strain, ε_CC _= 0.0054, ε_LL _= 0.0089, ε_RC _= 0.0072, ε_RL _= 0.0048 and ε_CL _= 0.0067. The LQC RMS differences, i.e. the residual of the polynomial mapping, averaged within the analytical model, were RMSx_R _= 0.030 mm in the radial direction, RMSx_C _= 0.017 mm in the circumferential direction, and RMSx_L _= 0.0004 mm in the longitudinal direction. The sensitivities to noise for each polynomial are plotted in Figure 2. The LQC polynomial yielded the smallest mean errors in the ideal situation and for a simulated noise with a standard deviation of 0.05 mm while the BLQ polynomial yielded the smallest mean error for the cases with 0.10 and 0.30 mm standard deviation of noise.

### *In vivo *evaluation

**Figure 3:**
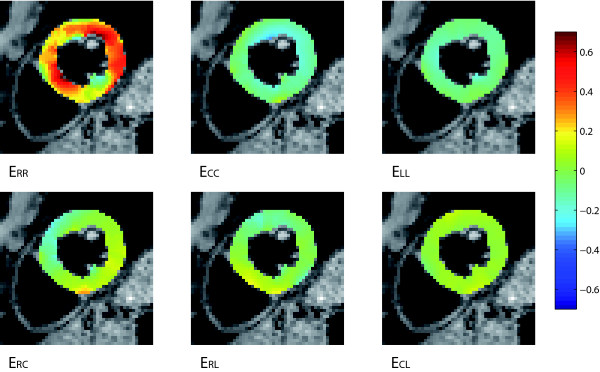
**Systolic strains**. All components of the end-systolic strain tensor at the mid-section of the 3D volume in a healthy volunteer estimated using the polynomial method.

**Figure 4:**
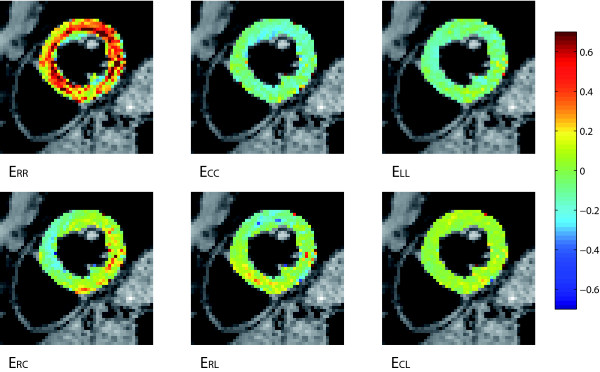
**Systolic strains**. All components of the end-systolic strain tensor at the mid-section of the 3D volume in a healthy volunteer estimated using a finite element method.

**Figure 5:**
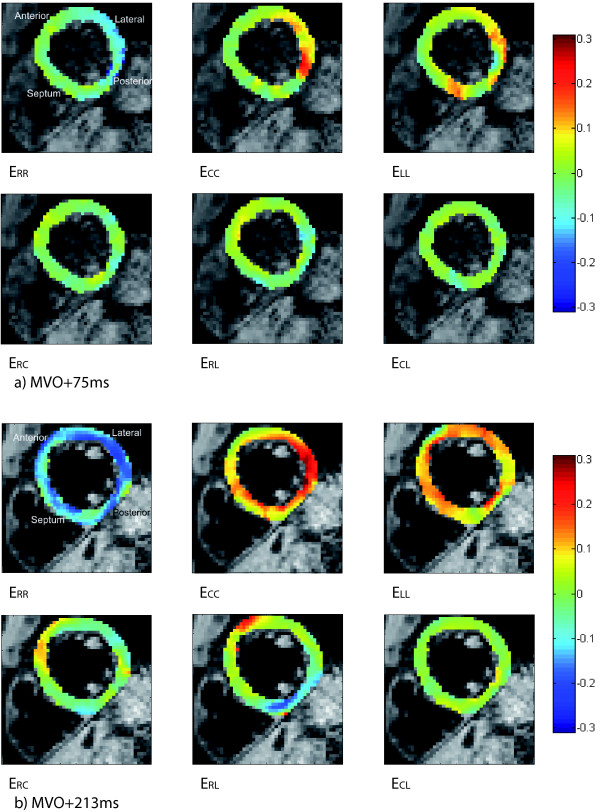
**Diastolic strains**. All components of the diastolic Lagrangian strain tensor at the mid-section of the 3D volume in a healthy volunteer. a) At 75 ms after MVO; b) At 213 ms after MVO.

**Figure 6:**
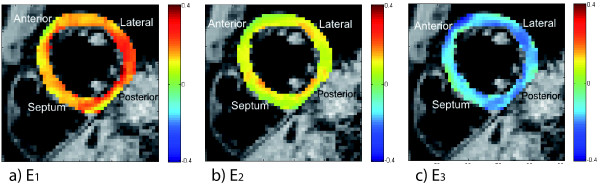
**Diastolic principal strains**. Diastolic principal strains at the mid-section of the 3D volume in a healthy volunteer, at 213 ms after MVO. a) E1; b) E2; c) E3.

The LQC and the BQC polynomials yielded the smallest maximum RMS differences in both systole and diastole. The results from the LQC polynomial are analyzed in further detail below. A mid-section (comprised of the short axis slices 5-7) is shown in Figure 3, 4, 5 and 6.

### Systole

The systolic strain components estimated using the polynomial method are shown in Figure 3. For comparison, Figure 4 shows the corresponding components estimated using a finite element method [17].

Maximum lengthening (up to 0.74) was found in the subendocardium in the septum and lateral free wall. Maximum shortening (down to -0.35) was found in the subendocardium and was essentially evenly distributed throughout the plane.

For all three directions, the lowest RMS values were found in septum and the highest values in the posterior wall. RMSx_R _was within the range 0.13 - 0.40 mm, RMSx_C _0.09 - 0.21 mm, and RMSx_L _0.09 - 0.24 mm.

### Diastole

All diastolic strain components at 75 ms (22% of the filling interval) and 213 ms (62%) after MVO at the mid-section of the 3D volume are shown in Figure 5.

The diastolic principal strains at 213 ms after MVO at the mid-section of the 3D volume are shown in Figure 6. Maximum lengthening (up to 0.35) was found in the postero-lateral wall while maximum shortening was essentially evenly distributed over the plane.

The highest RMS values were found in the posterior wall at 75 ms after MVO and in the anterior and posterior walls at 213 ms after MVO. The RMS values ranged from 0.07 mm to 0.40 mm for all directions at both diastolic times, except RMSx_R _at 75 ms after MVO which approached 0.50 mm in a small region in the posterior wall.

## Discussion

The proposed myocardial strain quantification method was initially developed for strain computation on data from a surgically implanted transmural bead array. However, this work shows that the method can be extended to be used with displacement data from displacement-encoded MRI.

This work uses a polynomial function to find a differentiable expression from the discrete displacement field. This polynomial function assumes continuous displacement in the myocardium, which reflects the connective properties of the myocardial tissue. This a priori information helps making the estimation more robust to noise.

The optimal order of the polynomial functions in equation (1) depends on the number of material points along each dimension, which in turn depends on the spatial resolution of the data, wall thickness and the sizes of the finite segments. Generally, the number of unknown constants in the polynomial should be less than the number of measured points along each dimension, which implies that a third order polynomial requires at least five measured material points along the corresponding dimension, a second order polynomial requires at least four points and a first order polynomial requires at least three points. Furthermore, to avoid an undetermined problem, the number of data points must be equal or greater than the number of coefficients in the minimization. This implies that the minimum number of required data points depends on the polynomial orders; BLQ requires 12 data point, BLC 16, LQC 24, and BQC 36. Hence, the polynomial order is limited by the number of included data points. This requirement was fulfilled for the *in vivo *evaluation; however, the margin for the BQC polynomial was small at some locations of the myocardium.

Four different polynomial orders were evaluated. The smallest absolute errors of the estimated strains in the analytical model in the presence of low noise were obtained with a linear-quadratic-cubic polynomial. In the subsequent *in vivo *validation, the *in vivo *results of the linear-quadratic-cubic polynomial were analyzed in detail. Given the incumbent spatial resolution, the restriction on **f**_R_(X_R_) was the wall thickness at the late diastolic time frame and the restrictions on **f**_C_(X_C_) and **f**_L_(X_L_) were the width and height of each segment, respectively. The width (π/6 radians) and height (7.5 mm) of each segment were kept small in order to resolve local variations of deformation.

The RMS differences between the acquired *in vivo *coordinates and the coordinates estimated using the polynomial fitting method reflect the accuracy of the polynomial fitting, giving a comprehension of the extent to which the coordinates estimated by the polynomial fit to the acquired *in vivo *coordinates. For both systolic and diastolic data, the RMS differences of the LQC polynomial were highest in the posterior wall. For the systolic data the region of highest RMS differences was close to the posterior papillary muscle, which might have caused locally irregular displacements. The RMS differences may furthermore be affected by the spatially varying signal-to-noise ratio in the DENSE measurement [18]. For the diastolic data, the region of highest RMS differences coincided with the region of thinnest wall. The present implementation of the method used the same polynomial order for each segment within the 3D volume. If a data set with large variations in wall thickness is considered, e.g. a 3D slab comprising an infarct with thin wall near the infarcted myocardium, the highest accuracy could be obtained by local adjustment of the size of the segments to the size of the infarcted region and adapting the polynomial order to wall thickness and segment width.

Systolic radial, circumferential and longitudinal strains, as well as systolic circumferential-longitudinal shear, show agreement with systolic strains previously reported for human myocardium [19,20]. E_RC_: We observed somewhat higher magnitudes of radial-circumferential shear strain than the results of Moore et al. [19], and we observed the lowest values in the anterior region of the myocardium while Moore et al. reported the lowest values in septum. E_RL_: The observed radial-longitudinal shear values fits within mean ± 2SD of the values reported by Moore et al. [19].

Diastolic function of the LV is determined by a complex sequence of many interrelated events and parameters including active relaxation, elastic recoil, passive filling characteristics, heart rate and inotropic state. Diastolic LV filling is a highly dynamic process with early and late transmitral inflows. Thus a detailed analysis of myocardial strain during diastole requires resolving the temporal process of diastolic filling.

The highest values of circumferential strain during the first 213 ms of diastolic filling were observed in the postero-lateral wall. The same quantitative behavior has been reported in previous studies of early diastolic strains in normal human hearts [21,22]. Radial strain, interpreted as wall thinning during diastole, was most apparent in the lateral wall, which also has been reported by others [21].

### Limitations

This work is limited to study the kinematics of the heart, focusing on strain. Strain should preferably be related to an unloaded, stress free reference configuration. Using *in vivo *data, there is no unloaded, stress free configuration of the heart. Instead, the reference configurations correspond to defined time points in the cardiac cycle. The strain presented in this, and similar articles within the field, therefore disregards the residual strain needed to study cardiac kinetics as opposed to kinematics.

*In vivo *validation of strain is challenging, which is why an analytical model was included in the validation. The analytical model can however never fully describe the cardiac kinematics. For *in vivo *estimation, the quality of the strain measurements is highly dependent on the quality of the underlying displacement data. While the polynomial fit reduces sensitivity-to-noise in the measurements, image artifacts or errors in the displacement measurements can deteriorate the strain estimates. Improving the DENSE acquisitions is an active field in the MRI research community, and strain analysis methods like the one presented in this paper will benefit directly from such improvements.

## Conclusions

In conclusion, the proposed method for strain estimation within a 3D myocardial volume yields accurate results when validated on an analytical model. The polynomial field is capable of resolving the measured material positions from the *in vivo *data, and the obtained *in vivo *strains agree with previously reported myocardial strains in normal human hearts.

## Competing interests

The authors declare that they have no competing interests.

## Authors' contributions

KK developed the analysis method, participated in the study design, carried out the analysis of the data, participated and coordinated the writing of the manuscript. HH participated in the study design, participated in the design of the data acquisition, participated in writing the manuscript. AS participated in the design of the data acquisition, participated in writing the manuscript. JE participated in the design of the data acquisition. NBI participated in the development of the analysis method, participated in the writing of the manuscript. TE participated in the study design, participated in the design of the data acquisition. MK participated in the design of the study. All authors read and approved the final manuscript.

## Pre-publication history

The pre-publication history for this paper can be accessed here:

http://www.biomedcentral.com/1471-2342/12/9/prepub
